# The Welfare Aggregation and Guidance (WAG) Tool: A New Method to Summarize Global Welfare Assessment Data for Equids

**DOI:** 10.3390/ani10040546

**Published:** 2020-03-25

**Authors:** Laura M. Kubasiewicz, João B. Rodrigues, Stuart L. Norris, Tamlin L. Watson, Karen Rickards, Nikki Bell, Andrew Judge, Zoe Raw, Faith A. Burden

**Affiliations:** The Donkey Sanctuary, Sidmouth, Devon EX10 0NU, UK; joao.rodrigues@thedonkeysanctuary.org.uk (J.B.R.); stuart.norris@thedonkeysanctuary.org.uk (S.L.N.); tamlin.watson@thedonkeysanctuary.org.uk (T.L.W.); karen.rickards@thedonkeysanctuary.org.uk (K.R.); nikki.bell@thedonkeysanctuary.org.uk (N.B.); andrew.judge@thedonkeysanctuary.org.uk (A.J.); zoe.raw@thedonkeysanctuary.org.uk (Z.R.); faith.burden@thedonkeysanctuary.org.uk (F.A.B.)

**Keywords:** welfare aggregation, equid welfare, methodology, resource allocation

## Abstract

**Simple Summary:**

Animal welfare can be considered in terms of health, the nutrition animals receive, the behavior of animals, the humans they interact with, their living environment, or working conditions. Each of these indicators can be measured in myriad ways. Consequently, welfare assessments tend to be lengthy, resulting in a wealth of data about each animal. There is, however, a need to report animal welfare concisely in order to compare and measure change. We propose a method to aggregate an existing questionnaire-based welfare assessment into five ‘grades’ to reflect the main components of animal welfare. We aim to provide a succinct way for stakeholders such as animal welfare charities to measure and report on welfare, aiding resource allocation, and enabling monitoring of the efficacy of intervention measures aimed at improving welfare conditions. In an assessment of the health and behavior of over 6000 equids across Europe and Asia, equids in Pakistan and India were found to have the poorest welfare levels. We recommend detailed assessments in these areas to identify the specific causes of the identified issues in order to guide the development of appropriate intervention schemes and, ultimately, improve equid welfare.

**Abstract:**

Animal welfare can be represented by an array of indicators. There is, however, increasing demand for concise welfare assessments that can be easily communicated and compared. Previous methods to aggregate welfare assessments have focused on livestock systems and produced a single welfare score, which may not represent all aspects of welfare. We propose an aggregation method for the recently developed Equid Assessment Research and Scoping (EARS) welfare assessment tool that results in grades for five welfare categories: housing conditions, working conditions, health, nutrition, and behavior. We overcome the problems associated with existing approaches by using a single aggregation method (decision trees) that incorporates the most important welfare indicators in a single step. The process aims to identify equids with the poorest welfare and aid decision-making when allocating resources. We demonstrate its application using a case study of over 6000 equids across Europe and Asia, where equids in India and Pakistan had the poorest welfare status in terms of health (respiratory disease and open wounds) and behavior (signs of fear and distress, and limb tethering practices). We recommend identification of the specific causes of these issues, using either existing detailed welfare data or through issue-specific assessments by an appropriate professional, to guide the development of appropriate interventions and, ultimately, improve equid welfare.

## 1. Introduction

The term ‘animal welfare’ can take on different meanings depending on the perspective of the observer [1]; from a veterinary focus on physical wellness, a behaviorist’s recognition of the mental state of the animal and manifestation as behavioral traits and, in terms of production or domesticated animals, the extent to which it lives a ‘natural’ life, (defined as the manifestation of genetic traits in breed and temperament [2]). Ultimately, good welfare can be viewed as the state in which an animal experiences a ‘good life’, which is generally accepted to include all three of these aspects [2,3]. As such, animal welfare is represented by an array, rather than a single measure, in order to reflect its multifaceted nature.

For production animals, whilst good welfare has inherent importance for an individual animal, adherence to acceptable standards is often driven by the potential for economic gain by stakeholders which, in turn, may be driven by incentives offered by governing bodies in the form of voluntary certification schemes [4], by concerns for public health or by compassion expressed by consumers, where animal products with high ethical standards are sought [5,6]. For working animals, one of the drivers leading to improved welfare is the potential economic gain from animals in good health [7]. In some cases, welfare may be improved due to the compassion exhibited by the owners themselves or by observers with the means to help [8]. For non-governmental organizations (NGOs) concerned with improving animal welfare, such help from observers often takes the form of donations, given with the intention that the NGO will work to improve the welfare status of animals in need. This approach requires that ‘animals in need’ can be identified in the first place, which is best achieved by conducting welfare assessments. Whilst recognition of the complexity of welfare is integral in the process of correctly identifying ‘animals in need’, the assessment and comparison of large groups requires that welfare can be aggregated into a digestible form [9]. 

Donkeys and mules have been a cornerstone of human existence and development for thousands of years [10] and continue to support human livelihoods through their role as pack animals in agriculture and industry, traction animals on farmland and in forests, and in the transportation of humans for both necessity in isolated areas and for tourism [10,11,12,13]. There are an estimated 54 million donkeys and mules worldwide, with approximately 43% of these found in Asia [14]. Donkeys, in particular, have long been perceived as having low status and are often treated as expendable [15]. However, a high proportion of donkey owners live below the poverty line and are completely reliant on their ‘beasts of burden’ as their main source of income [16]. As well as the inherent suffering that ill-treatment and poor welfare inflicts on the equids themselves, an equid that is unable to work will disproportionately and negatively impact upon human livelihoods, as recognized by the United Nations (UK) [17]. The ability of any stakeholder to assess, influence, or advocate for equid welfare first relies on their ability to accurately assess, monitor and report on it. It is therefore imperative that the assessment of equid welfare is conducted using a systematic approach that yields consistent and comparable datasets. 

The aggregation of welfare assessments into a single welfare score is a contentious subject [18]; single measures of welfare may not be sufficient to reflect the multifarious nature of welfare as a whole [19,20] and the process may combine aspects of welfare that exist on different value scales [19]. Whilst existing welfare aggregation methods such as the Welfare Quality Assessment^®^ (WQA) [21] and Animal Needs Index (ANI-35) [22] are widely used across Europe, these methods are aimed exclusively at the assessment of livestock, with the ANI-35 used solely to address housing conditions. Whilst the WQA overcomes the pitfalls of individual aggregation methods by using a suite of different techniques over three stages of integration, the process is complex and it may be difficult to interpret in terms of the measures of welfare at which improvements should be aimed [23].

We propose a transparent, single-step method of aggregation that results in separate ‘grades’ for five welfare categories: (1) health, (2) behavior, (3) working conditions, (4) living environment, and (5) nutrition. These categories were used as a basis for grouping questions in the Equid Assessment Research and Scoping (EARS) tool [24], a questionnaire-based welfare assessment on which the aggregation method is based, and reflect the four physical (functional) domains of the five domains model [25], with the fifth, ‘affective experience’, incorporated into categories where applicable. The EARS tool incorporates a range of welfare indicators to enable the assessment of equids in any environment, including those in production, working animals, companion or therapy equids, and free-roaming equids. Our approach aims to provide a tool for organizations that need to identify differences in welfare states across groups of equids, tailor actions to improve equid welfare and monitor change. 

Specifically, the proposed aggregation method will enable the following:

- The identification of spatial trends across five categories of welfare to allow the identification of equids with the poorest welfare (or ‘greatest need’).
- The identification of temporal trends in welfare, which will enable intervention work to be monitored and evaluated.
- The provision of guidance on which aspects of welfare require further, more detailed, assessment and which resources, aimed at improving welfare, may be required.

First, we review existing aggregation processes, then outline the method for the proposed new system, and demonstrate its use via a case study comparing welfare between equids in Europe and Asia in order to identify those with the poorest welfare. Finally, we make recommendations to guide decisions on the allocation of resources to target the equids in greatest need of intervention and to make the most effective improvements to equid welfare. 

## 2. Materials and Methods

### 2.1. The Development Process

Welfare aggregation methods can be grouped into several types, with selection based on the requirements of stakeholders. They include individual non-standardized assessments used to inform policy [26,27]; threshold approaches, where minimum requirements are set and checked during certification reviews; and standardized scoring systems based on ranked and summed (or averaged) scores of scaled indicators, which can be used for risk assessments and monitoring [28]. As previous methods are reviewed thoroughly in Botreau, et al. [28], we will instead focus on how the main caveats and pitfalls of existing methods have been tackled during development of the current process. 

#### 2.1.1. A Single Welfare Score?

Single measures of welfare provide the most efficient and communicable way to monitor and report change. Whilst methods to produce single welfare measures have been utilized successfully in the past [18,21], these methods are aimed at specific environments (i.e., housing systems in livestock farms) and may require the use of an advisor to highlight aspects of the assessment requiring attention from the farm manager, rather than being taken at face-value [21]. 

The EARS tool, however, includes assessments for animals in a number of different environments, including animals in production farms, working equids, and companion animals [24]; welfare assessments are required to guide resource allocation for NGOs, for which each of the welfare categories are typically considered individually. Specific welfare improvements will be expected from particular interventions, which can also be linked to specific welfare categories. For our purposes, therefore, welfare is presented as the aggregated scores of the five broad categories of health, behavior, working conditions, living environment and nutrition.

#### 2.1.2. Do (and Should) Good Scores Compensate for Bad Ones?

One of the most commonly cited issues with aggregating welfare indicators is that good welfare in one aspect compensates for poor welfare in another [28,29,30]. This problem can be considered at two scales: an animal obtaining an equal number of ‘good’ and ‘poor’ assessments for different categories of welfare, or a group consisting of an equal proportion of animals with ‘good’ and ‘poor’ welfare scores, will obtain the same score as a unit obtaining all ‘average’ scores [31]. 

##### Single Animal: Within and Between Categories

Whether or not different welfare categories should compensate for each other draws on the ethical standpoints of utilitarianism and deontology and remains under debate (see Botreau et al. [24] for full discussion). To avoid the issue, Botreau et al. [31] suggest stopping the aggregation process at criterion (or category) level and thus only allowing compensation between indicators that interact. For the current process, whilst categories are kept separate, each are measured by several questions that represent the same dimension but not necessarily the same problem. Further measures have therefore been employed to address the issue; namely, keeping the number of questions to a minimum [19,23] and ensuring all questions within a category are either all animal-based (behavior, health, and nutrition) or environment-based (working conditions and living environment) so that the type of impact they reflect is comparable [28].

##### Between Animals

One of the main concerns when allocating limited resources to improve welfare is how to balance the needs of many animals with average welfare with a few animals with extremely poor welfare [30]. Whilst the allocation of resources between these two scenarios is a management decision, the requirements of a welfare aggregation process are to highlight when these scenarios exist. 

In the current process, grades for an assessed group of equids will be highlighted with an asterisk when 15% or more of the animals received at least one ‘red’ (poor welfare) answer. This approach allows us to differentiate between populations with the same grade based on the presence of a higher proportion of animals with very poor welfare and prevents these animals from being ‘hidden’ by the presence of the same proportion of animals with excellent welfare. Ideally, all animals with a ‘red’ score would be highlighted in the process. However, as the current process is aimed at guiding resource allocation from NGOs, the 15% threshold provides an acknowledgment that those resources are inherently limited. The threshold of 15% fits with the ‘15% rule’ used in the WQA [21], which is in accordance with the ‘priority view’ of Derek Parfit, a British moral philosopher, whereby all individuals are considered within any given assessment but extra weight is given to those who are worse-off; a view that is widespread within the welfare aggregation literature [30,32,33].

#### 2.1.3. Animal-Based or Environment-Based Measures?

Environment-based measures are considered less relevant to welfare than direct animal-based measures in many cases [34,35] as they provide a ‘risk assessment but not an evaluation of the actual welfare state of animals’ [19]. For some categories, however, environment-based measures may indicate the cause of poor welfare or correlate strongly with an animal-based measure [21]. The EARS tool contains both animal and environment-based measures, with the majority measures in the health and behavior categories belonging to the former; measures in the nutrition, living environment, and working conditions categories belonging to the latter [24]. We, therefore, allowed both types of measures, with the selection based on which were most indicative of (or correlated most strongly with) welfare.

#### 2.1.4. Is the Process Transparent and Easily Explained to Stakeholders?

One of the main requirements of an aggregation system is transparency in the process [18], particularly considering the unavoidable need for opinion-based decisions on which factors to include, how to scale or rank answers in terms of the level of welfare they reflect and, ultimately, what constitutes good or poor welfare and requires attention [28,30]. To maintain transparency, the process is limited to a single aggregation step, from measures to categories. As the aims of the process are to guide operational decision-making and allow monitoring of the impact of intervention work, the production of five scores reflecting the broad (and different) aspects of welfare fulfils these aims as a welfare indicator can be selected to match to operational work being monitored. 

#### 2.1.5. Do Important Attributes Have Enough Influence?

This question largely arises from the uneven design in previous aggregation methods. In the WQA, for example, the requirement to include an as many welfare measures as possible into the process results in differing numbers of measures per category, incorporated using a suite of integration methods [31], which have variable influence on the final welfare score [23]. The current approach employs a standard, one-step aggregation method, with an equal number of questions per category. All questions, therefore, have the same influence on the final welfare grade. 

Whilst this approach makes the process transparent and easy to interpret, in practice all four welfare indicators are unlikely to have the same impact on welfare. By using expert opinion (see methods) to rank all available questions and selecting the four most influential indicators within each category, we have gone some way to account for this limitation. Whilst the simplicity of the process allows transparency and ease of understanding, this decision does limit its interpretive power. For this reason, the results indicate a focus for further assessment and must be used as one part of a wider decision-making process, rather than as the sole platform upon which to build an intervention program. 

### 2.2. The WAG Process

#### 2.2.1. Category Selection

The welfare aggregation protocol is based on the Equid Assessment Research and Scoping (EARS) tool; a questionnaire-based method recently developed by The Donkey Sanctuary (TDS) to assess equid welfare in all contexts (i.e., working, domestic, production or feral), in a standardized way. Individual equids and their environment are assessed via a series of ‘yes/no’ and ordinal questions that were selected as having a significant influence on welfare. Questions are available in a series of protocols tailored to the requirements of the assessor or project. Details of the development process can be found in Raw et al. [24]. 

For the WAG process, questions were divided into categories based on the physical/functional domains of The Five Domains model i.e., Nutrition, Environment, and Health and Behavior [25]; whilst the mental state domain, ‘affective experience’, is incorporated into the behavior category via the questions ‘What is the general attitude of the equid at a distance?’ and ‘Please indicate signs of fear and distress’. The environment domain could, potentially, be represented by two conditions; the environment whilst working and the environment outside of work (including times outside of the working period or the living conditions of non-working equids, such as feral equids or those kept for production). An aim of the aggregation system was to allow stakeholders to focus intervention work towards animals with the poorest levels of welfare, including specific interventions aimed at the working or living environment. To meet this aim, we present working conditions and living environment as separate categories. 

#### 2.2.2. Question Selection

The full list of EARS questions within each category were sent to at least two experts in the respective field of each category. Experts were selected based on their professional specialisms and level of knowledge of equid welfare. Experts included equid veterinary surgeons, welfare professionals and behavior professionals. Experts were asked to (a) rank questions in terms of which are most indicative of welfare, or which have the most impact on welfare if the worst-case scenario is realized. Experts could assign more than one question to a particular ranking, but were asked to minimize this practice as much as possible; and (b) rank the answers to each question on an ordinal, three-point, colorimetric scale, where answers indicating the poorest levels of welfare were designated as red, those of medium indication were designated amber, and those indicating the best level of welfare were designated as green; each color could be assigned to more than one answer per question if needed. Some questions include an ‘other’ option (Table 1; Tables S1 and S2), where the extra data provided could be assessed and classified under the same colorimetric scale, as needed on a project by project basis. Experts were asked to provide separate rankings for adults (at least one year old) and for foals. For inclusion in the process, all questions had to meet the following guidelines:

##### Questions must be Present in all EARS Assessment Protocols

The EARS process includes a selection of protocols, from an overarching ‘scoping’ protocol, which is carried out in during all assessments, with additional, in-depth, protocols used where appropriate to investigate specific health or environmental conditions. Whilst more detailed, specialist knowledge may give a more comprehensive assessment of welfare, the aim of the aggregation process is to give a broad overview of welfare trends. To ensure that it is possible to collect sufficient data to enable broad scale trends in welfare to be identified, questions were selected from the scoping protocol only. The process has, however, been designed to be as transparent as possible, therefore results can be examined in detail later in the process, and targeted examinations recommended to provide further detail during subsequent stages of assessment.

##### It must be Possible to Answer all Four Questions for each Assessment

Question that are linked to another (for example, the EARS working conditions category contains questions about harness suitability, which are only included when the assessor indicates that the animal is wearing a harness). In these cases, the question will not be included unless a suitable replacement is available to cover the instance where they cannot be answered (Tables S1 and S2). ‘Suitable replacement’ is defined as a question with a ranking within 1 point of the original question.

##### Questions must Contain at least One ‘Green’ and One ‘Red’ Answer in order to Represent a Range of Welfare Conditions

Answers designated as ‘amber’ allow a greater separation of scores, therefore finer detail when analyzing results but are not integral to the process. It was decided, however, that all questions should contain options that reflect the best- and worst-case scenario.

For each category, rankings from experts were compiled and the top four questions were selected. Where discrepancies existed in scores between experts that affected the final question selection, experts were asked to discuss their answers until a consensus was reached. If a consensus could not be reached, scores were averaged (although this did not occur in practice). As the behavior and health categories are used in the case study, the question selections for these categories are presented as an example in Table 1 (question selections for all categories are provided in Tables S1 and S2). 

#### 2.2.3. Aggregation Step 1: Assigning Category Scores to Individuals

**Figure 1:**
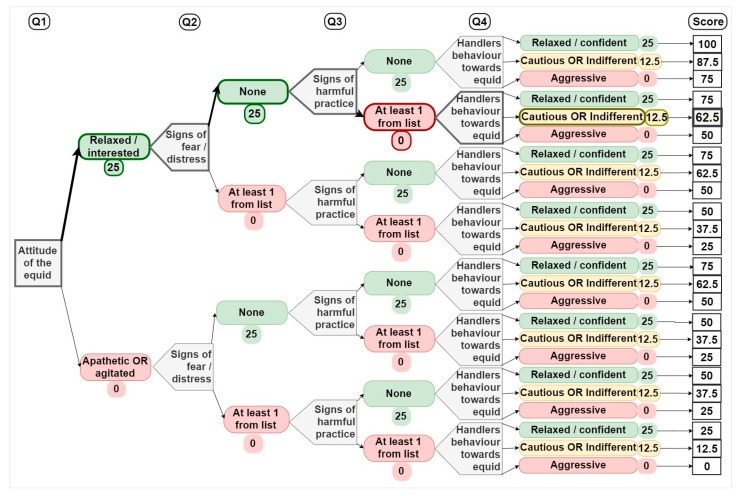
Example decision-tree for the behavior category. Each question scores a maximum of 25 points. Points are summed for all four questions to give the category score. In the example above (indicated by bold arrows and boxes), an equid that was assigned a ‘green’ answer for Q1 and Q2, red for Q3, and amber for Q4 would score a total of 62.5 points. The full list of answers for Q2 and Q3 are provided in Table 1.

The four questions for each category were combined using a decision tree approach adapted from Welfare Quality^®^ [21]; individuals were only included if data were available for all four of the questions in the decision tree as follows: The answers for each question were grouped according to color and a standard score was applied to each color, where green = 25, amber = 12.5, and red = 0; the scores for all four questions were then summed to give a Category Score ranging from 0 to 100 (Figure 1). For single choice questions, a single score was given based on color group the answer falls in to; for multiple choice questions, a single score was given based on the lowest scoring answer. This rule is based on the logic that the presence of negative traits (indicated by the presence of an indicator of poor welfare i.e., a ‘red’ answer) should not be outweighed or moderated by the presence of accompanying positive traits. There are currently two exceptions to this rule; the question ‘describe the main diet of the equid’ and ‘describe the foal’s access to milk’ in the nutrition section for adults and foals, respectively (Tables S1 and S2). These questions differ from the others as the presence of positive welfare indicators supersedes the presence of a poor one, hence a single score based on the highest scoring answer is applied for these questions.

#### 2.2.4. Aggregation Step 2: Assigning Category Grades to Individuals

As individual scores are a sum of four static values, they naturally fall into bands. At this stage, the application of a ‘grade’ to each of these bands allows for a clearer distinction between the cumulative percent values required in the next stage of aggregation, and the scores themselves. Grades also provide an easily understood metric for use in reporting, particularly when up to five grades may be presented for one group of equids. 

Each individual score is assigned a grade as follows: A = 100 to 90; B = 89 to 80; C = 79 to 70; and so on, through to J = 10 to 1. For the purpose of comparing broad scale trends (e.g., welfare levels between countries or regions, or through time), the distribution of individual grades can be considered at this stage. This approach is informative when a large dataset is available, as it allows for the maximum use of data and identification of any anomalies for further investigation. 

#### 2.2.5. Aggregation Step 3: Combining Grades

An overall grade is applied to the selected group of animals using the 15% rule from Welfare Quality^®^ [21]. Following this rule, the overall grade is assigned as the lowest grade assigned to a cumulative total of at least 15% of the animals in the group, where percentages are accumulated from the worst grade (J) to the best (A; see Table 2).

#### 2.2.6. Aggregation Step 4: Highlighting Unequal Variance

The aggregation process involves a sum of scores, so an animal with a medium score for all four questions in the process will obtain the same grade as an animal with two good (green) and two poor (red) responses. To enable differentiation between groups containing animals with different combinations of scores, the grade for a group will be highlighted with an asterisk when 15% or more of the animals received at least one ‘red’ answer. 

All data processing and analyses were performed in R version 3.6.1 [36] through R studio version 1.2.1335 [37] using the following packages: dplyr, forcats, ggplot2, rowr, stringr, purrr, readrr, tibble, tidyr, and tidyverse [38,39,40,41,42,43,44,45,46,47]. 

### 2.3. Comparison of Behavior and Health in Countries within Asia and Europe

To review the ability of the process to identify equids with the poorest welfare and guide resource allocation, the aggregation process was carried out on assessments from several countries within Europe and Asia. These locations were selected based on the division of operational work within TDS, which was originally put in place to reflect anecdotal evidence for differences in both the overall level of welfare and the specific needs within each region. 

An existing TDS dataset consisting of 6490 EARS assessments was used; assessments were carried out between September 2018 and August 2019, with 5266 and 5870 complete assessments for behavior and health, respectively, across the two regions (Table 3). As a separate assessment for behavior are available for foals and adults (see Tables S1 and S2), assessments were limited to equids at least one year old. Equids within the assessment will be described in terms of demographics and roles to provide context for the current analysis. Whilst a full analysis using the WAG process is possible using each of these metrics to further refine guidance for resource allocation (depending on the requirements of the stakeholder), analysis in the current case study will be limited to locations for demonstrative purposes.

All EARS assessments were carried out by fully trained assessors (see Raw et al. [24] for full methodology). For the aggregation process, welfare grades were calculated for the behavior and health categories for each of the two regions, as well as for each individual country. This initial, broad assessment is followed by a more detailed examination of the country with the poorest welfare grades to identify areas for further assessment and to guide suggestions for intervention work. We limited the analysis to the health and behavior categories for two reasons. Firstly, the amount of data available for these categories was high, enabling the spatial comparison. Secondly, limiting the example to two of the categories gave us scope to fully demonstrate the practical use of the WAG process by presenting an in-depth post-aggregation evaluation.

## 3. Results and Discussion

### 3.1. Demographics

Within Asia, the majority of equids in the cohort worked as draft or pack animals (98%) with the remaining equids being in production farms (2%). In Europe, equids were mainly companion or sanctuary animals (89%), with the remaining equids working as draft or pack animals (7%) or assessed at production farms (3%) or religious festivals (1%; see Table 4 and Table S3 for a breakdown of roles per country). 

In Asia, 20% of the cohort were donkeys, 47% were hybrids (mules and hinnies) and 32% were horses. In Europe the breakdown per species was 86% donkeys, 13% hybrids, and less than 1% horses. In Asia, 73% of equids were male and 27% were female. In Europe, 63% were male and 37% were female (see Tables S4 and S5 for a breakdown of species and sexes per country).

### 3.2. Assessment of Broad Scale Trends

Overall, both health and behavior were poorer in Asia (health = D*; Behavior = E*) than in Europe (health = C; Behavior = B). Within Asia, health and behavior grades were poorest in India and Pakistan, with grades of E* and G* for health, and an H* and E* for behavior, respectively. Nepal and China were also highlighted as obtaining above the threshold level of 15% red scores for health, which did not occur for any of the countries in Europe. The highest health grades of B were obtained in Ireland and Spain. For behavior, the UK obtained a B grade, whilst all other countries of those tested in Europe obtained a C* (Table 5).

**Figure 2:**
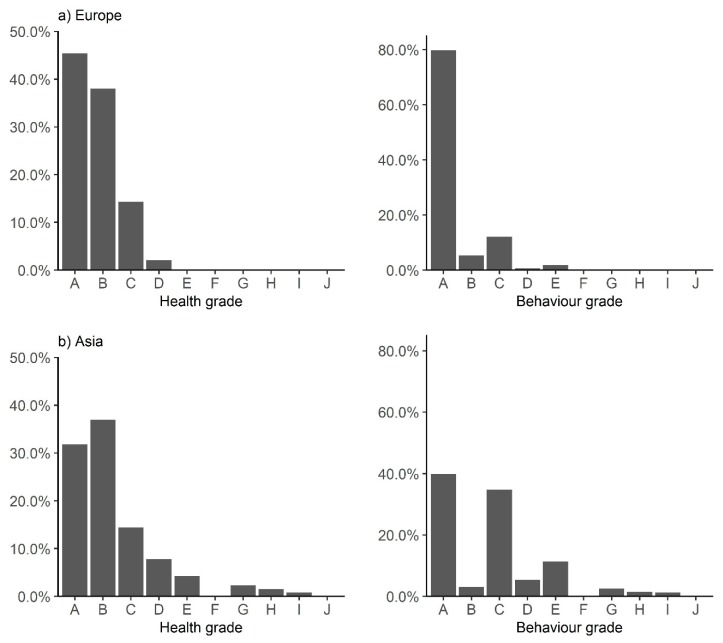
Percentage of animals that obtained each welfare grade for health and behavior in (**a**) Europe and (**b**) Asia.

A review of the distribution of individual grades (per animal) within each region (Figure 2) suggests that, whilst both behavior and health are poorest in Asia, there is a wide distribution of grades in both regions with a small proportion of animals obtaining grades D for health and E for behavior in Europe. These scores indicate that, whilst the greatest need for intervention work resides in Asia, there is scope for welfare improvements in both regions. The majority of equids in Europe are companion or sanctuary animals whilst a proportion of these may have been recently removed from harmful conditions, resulting in poor welfare grades, a full assessment of the drivers of welfare is recommended to identify equids requiring intervention. However, to meet our aim of identifying regions and countries in greatest need, the current case study will focus on Asia.

### 3.3. Assessment of Drivers of Poor Welfare

**Figure 3:**
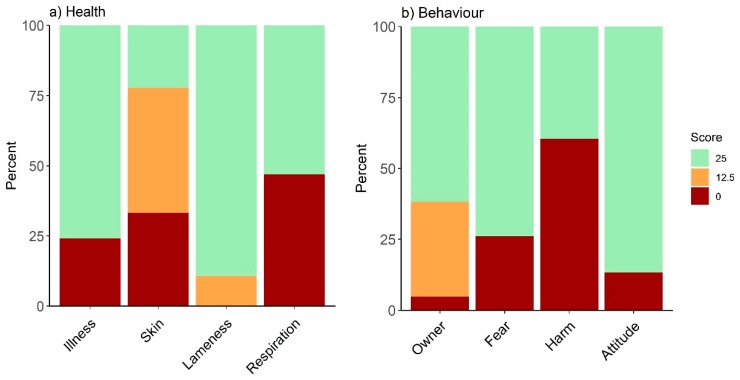
Percentage of responses obtaining each of the available scores per question for the (**a**) health and (**b**) behavior decision trees for Pakistan.

To understand the drivers of poor welfare scores for behavior and health, the proportion of individuals with good, medium, and poor scores for each question were examined. As the country with the poorest grades for both categories, results are presented for Pakistan (Figure 3). Within the health category, 48% of animals obtained a ‘red’ score for ‘signs of respiratory disease’ (Figure 3a); of these, 46% displayed nasal discharge and 54% displayed eye discharge, with 28% of these equids displaying signs of both. In total, 78% of animals received an amber or red score for ‘signs of skin system alteration’; of which, 97% of red responses were due to open wounds, and 90% of amber responses were due in equal part to scars and alopecia. For behavior, equids in this region presented more red responses for ‘signs of both fear and distress’ and ‘evidence of harmful practice’ than the other issues examined (26% and 60%, respectively), whilst owner behavior received a notable proportion of amber responses (33%; Figure 3b). Fear and distress were predominantly expressed as ‘showing whites of the eyes’ (23%) and ‘head shyness’ (22%). Harmful practices were predominantly observed as ‘the presences of signs of limb tethering or hobbling’ (75% of red responses), whilst owner behavior was recorded as ‘assertive or indifferent’ or ‘cautious or fearful’, accounting for 47% and 53% of amber responses, respectively.

### 3.4. Recommendations Based on the WAG Process

Of the two regions under assessment, welfare was poorest in Asia in terms of both health and behavior. Of the countries assessed within Asia, equids with the poorest levels of welfare were found in India and Pakistan for both categories. Examination of all existing detailed welfare data, where available, is recommended as a priority for these countries. As a case example, specific recommendations for the development of interventions aimed towards equids in Pakistan are as follows:

All of the equids under assessment in Pakistan worked as draft or pack animals. Twenty-seven percent worked in brick kilns or coal mines (Table S3); of these, 65% obtained a ‘red’ score for respiratory disease, as opposed to 40% of the remaining cohort. Whilst it seems likely that dust may be a contributing factor [48], further investigation is required to identify whether the cause of poor respiratory health is environmental or infectious. If an environmental cause is confirmed, social research should be conducted to understand the lives and options of equid owners, particularly in brick kilns and coal mines, to inform intervention work tailored towards helping people minimize equid exposure to harmful dust whenever possible. 

In terms of wounds, more detailed assessment by appropriately qualified professionals are suggested to identify the main causes. The working nature of the equids may focus these assessments on harness fit, farriery and handling. A large proportion of equids had signs of tethering (which restricts freedom and suggests they are likely to be kept in close quarters), which may increase susceptibility to animal attacks (both con- and allo-specific). 

In total, 55% of equids worked to transport goods to market (Table S3), which may result in vehicle collisions. Identification of the cause of skin disorders leading to alopecia including examination for ectoparasites, insect bites, or bacterial infection is also recommended and would need to be undertaken by a veterinarian. These assessments can then be used to tailor intervention programs specifically towards the prevention of wounds and skin disorders.

It would be reasonable to deduce that the fear and distress exhibited is associated with aversive anthropogenic activities [49] such as poor handling or debilitating work demands, as supported by the high proportion of ‘indifferent’ or ‘fearful’ owners, or by harmful practice, as demonstrated by the high proportion of equids with signs of tethering or hobbling. The human-equid partnership is built over time and based on repeated interactions which define expectations on both sides [50]. Positive interactions lead to reassurance and confident association [49,50,51]. 

As a proportion of the behavioral and health concerns highlighted in this study are likely to be anthropogenic, they could conceivably be overcome by culturally sensitive, bespoke educational programs, and training once understanding is gained about the internal and external factors driving the human behavior within each context. For example, safer tethering practices could be introduced and alternatives explored. Interventions regarding handling and equid management could be aimed at owners, though improved training for professionals and paraprofessionals would ensure a strong, consistent message is developed at all levels. Any interventions should be subject to a rigorous monitoring and evaluation system to ensure objectives are achieved [52] in accordance with the strategic directives of the programs within each organization.

## 4. Conclusions

We provide a transparent, standardized process for identifying trends in equid welfare and monitoring change through time. By adopting a single-step approach and describing five categories of welfare, we avoid some of the main pitfalls of existing aggregation methods whilst maintaining focus of the operational use of the tool. 

The simplicity of the current method ensures that results are transparent and easily interpreted. The use of decision trees means that grades and scores are traceable back to their components during analysis of the equids found to be in greatest need. This simplicity does, however, mean that not all aspects of welfare are incorporated into the process. Whilst questions were selected based on their indicative value for each welfare category to limit the impact of this issue as much as possible, we recommend that the results of the welfare aggregation process are scrutinized alongside any additional welfare information that is currently available for the group. As the category grades focus attention on a small number of equids in greatest need, more detailed assessment would be manageable and informative. 

Welfare grades form an important part of any decision-making process regarding how to most effectively utilize funds, target interventions, and create change where it is most needed. However, we acknowledge that there are many other factors which may influence resource allocation and programming. Although the decision-making process itself is beyond the scope of this paper, welfare, coupled with population size, could be considered alongside other strategic elements such as operational feasibility (regional conflict status, presence of partner organizations etc.) and potential impact (presence of social structure that enables change), during project development.

## Figures and Tables

**Table 1:** Questions selected for the health and behavior categories.

	Question	‘Green’ Responses	‘Amber’ Responses	‘Red’ Responses
Health	Are there any signs of skin system alterations? ^∆^	no	Scars; alopecia; swellings	Open wounds; sarcoids
	Is the equid lame?	no lameness	yes (intermittently or consistently lame)	yes, non-weight bearing; severely lame/unable to walk
	Please indicate obvious signs of illness ^∆^	no signs present		nasal and/or eye discharge; signs of diarrhea; significant discharge from penis or vulva; abdominal pain
	Is the equids coat healthy?	Yes		no
Behavior	General attitude of the equid at a distance? ^∆^	at ease-relaxed, calm and/or resting; alert and actively interested in surroundings		apathetic, depressed, withdrawn; agitated, aggressive, hyper-reactive/vigilant
	Please indicate signs of fear and distress present ^∆^	no signs of fear and distress present		showing the whites of the eyes; unpredictable or sudden movements; sudden startle responses; aggressive behavior; trembling; head shyness; completely withdrawn/shut down
	Presence of signs of harmful practices? ^∆^	no		signs of hot brand, firing; limb tethering or hobbling; amputations or mutilations; use of live serreta or similar
	Owner’s/user’s/handler’s interaction when holding the equid? ^∆^ *	relaxed and confident	assertive/indifferent	aggressive
			cautious/fearful	
	Is the equid with other animal(s)? **	yes, physical contact	yes, visual contact	no

**^∆^** Indicates questions that include an option for ‘other’, for which data can be examined individually and classified under the colorimetric scale. * Indicates the main question in the behavior decision tree; ** Indicates an alternative question for when the animal’s owner is not present, to fulfil rule no. 2 (see ‘question selection’ section)

**Table 2:** Selection of final welfare grades for two different scenarios, using the 15% rule = [26]. The first column gives the percentage of animals within the group that received each grade after the third stage of aggregation (individual grading). The final welfare grade for the group is indicated as the first grade at which at least 15% of the individuals received that grade or a lower one, indicated by the cumulative percentage taken from worst to best grade.

Group	% of Animals that Received Score	Cumulative %	Final Grade
**Group A**			
A =	27%	100%	
B =	39%	73%	
C =	15%	34%	
**D =**	9%	**19%**	**←Final grade = D**
E =	2%	10%	
F =	5%	8%	
G =	0%	3%	
H=	2%	3%	
I =	1%	1%	
J =	0%	0%	
**Group B**			
A =	30%	100%	
B =	39%	70%	
**C =**	19%	**31%**	**←Final grade = C**
D =	3%	12%	
E =	6%	9%	
F =	2%	3%	
G =	0%	1%	
H=	1%	1%	
I =	0%	0%	
J =	0%	0%	

**Table 3:** The number of Equid Assessment Research and Scoping (EARS) assessments completed in each country, grouped by region. Numbers in the ‘behavior’ and ‘health’ columns indicate the assessments that provided sufficient data to complete the behavior and health decision trees, respectively.

Region	Country	Total No. Equids Assessed	Health	Behavior
Asia	China	61	61	0
	India	103	73	39
	Nepal	2557	2459	1850
	Pakistan	635	514	591
Europe	Cyprus	52	52	52
	Greece	231	164	121
	Ireland	70	68	70
	Italy	120	113	83
	Romania	36	36	31
	Spain	38	38	18
	United Kingdom	2658	2523	2535
**All**		6561	6101	5390

**Table 4:** Role of the equids assessed in each region and country. For working animals, the place or type of work is provided as a list. The percentage (Percent of equids) and number (n) of equids in each specified role per country is provided.

	Countries	Equid Role	Place or Type of Work (if Applicable)	Percent of Equids	(n)
Asia	China	Production animal	--	100	(61)
	India	Draft or pack animal	Brick kilns; construction sites	100	(103)
	Nepal	Draft or pack animal	Brick kilns	100	(2557)
	Pakistan	Draft or pack animal	Brick kiln; coal mines; farm traction, transport of goods to market	100	(635)
Europe	Cyprus	Companion/sanctuary animal	--	100	(52)
	Greece	Draft or pack animal	Construction site; tourism (riding)	92	(213)
		Companion/sanctuary animal	--	8	(18)
	Ireland	Companion/sanctuary animal	--	100	(70)
	Italy	Production animal	--	81	(97)
		Companion/sanctuary animal	--	19	(23)
	Romania	Companion/sanctuary animal	--	94	(34)
		Draft or pack animal	Transport of goods to market	6	(2)
	Spain	Other work	Religious festival	100	(38)
	United Kingdom	Companion/sanctuary animal	--	100	(2658)

**Table 5:** Final grades obtained for each region for the welfare categories ‘behavior’ and ‘health’. Asterisks indicate that at least 15% of the animals in that region received a poor (red) assessment for one of the questions in the decision tree.

Region	Country	Local Grade	
		Health	Behavior
Asia	China	C*	--
	India	E*	H*
	Nepal	C*	D*
	Pakistan	G*	E*
Europe	Cyprus	C	C*
	Greece	C	C*
	Italy	C	C*
	Ireland	B	C*
	Romania	C	C*
	Spain	B	C*
	United Kingdom	C	B

## Supplementary Materials

The following are available online at https://www.mdpi.com/2076-2615/10/4/546/s1; Table S1: Questions and responses for the welfare categories of health, behavior, nutrition, working conditions and living environment categories, Table S2: Foal-specific questions for the nutrition and behaviour categories. Table S3: Role of the equids assessed in each region and country, Table S4: Percentage (%) and number (n) of each species of the equids assessed, Table S5: Percentage (%) and number (n) of each sex of the equids assessed.
