# Supplementary material for: The Welfare Aggregation and Guidance (WAG) Tool: A New Method to Summarize Global Welfare Assessment Data for Equids
Source: Animals (Basel). 2020 Mar 25;10(4):546. doi: 10.3390/ani10040546 (PMC7222376; doi:10.3390/ani10040546)
Supplement: Supplementary file 1 [file animals-10-00546-s001.pdf]

Table S1: Questions and responses for the welfare categories of health, behaviour, nutrition, working conditions and housing conditions categories. The questions indicated with asterisks represent the main question in the decision tree (*), and an alternative question (**) for conditions where it is not possible to answer the main question.

Category	Question	'Green' responses	'Amber' responses	'Red' responses
General health	**Are there any signs of skin system alterations?**	no	yes, scars<br>yes, alopecia<br>yes, swellings	yes, open wounds<br>yes, sarcoids
	**Is the equid lame?**	no lameness	yes (intermittently or consistently lame)	yes, non-weight bearing<br>yes (severely lame/unable to walk)
	**Please indicate obvious signs of illness**	no signs present		nasal and / or eye discharge<br>signs of diarrhoea<br>significant discharge from penis or vulva<br>abdominal pain
	**Is the equids coat healthy?**	Yes		No
Behaviour	**General attitude of the equid at a distance?**	at ease - relaxed, calm and/or resting<br>alert and actively interested in surroundings		apathetic, depressed, withdrawn<br>agitated, aggressive, hyper-reactive/vigilant
	**Please indicate signs of fear and distress present**	no signs of fear and distress present		showing the whites of the eyes<br>unpredictable or sudden movements<br>sudden startle responses when standing quietly<br>aggressive behaviour<br>trembling or head shyness<br>completely withdrawn/shut down
	**Presence of signs of harmful practices?**	no		signs of hot brand, firing<br>signs of limb tethering or hobbling<br>signs of amputations or mutilations<br>signs of use of live serreta or similar
	*** Owner's/user's/handler's interaction when holding the equid?**	relaxed and confident	assertive / indifferent<br>cautious/fearful	aggressive
	** **Is the equid with other animal(s)?**	yes, physical contact	yes, visual contact	No
Nutrition	**Body condition?**	ideal	thin/moderate<br>fat	very thin/poor<br>very fat/obese
	**For how long is fibre available?**	18-24	14-18	Below 14
	**Is the equid getting an appropriate diet?**	Chopped fibre, chaff, other prepared fibre<br>Grass or browse<br>Hay or haylage or straw/stover<br>vitamins / mineral	legumes/pulses<br>creep feed	Silage<br>cereal grains<br>Sugar based products
	*** Access to clean water during working period**	free access to clean water	free access to partially dirty water<br>limited access to clean water	Free access to dirty water<br>limited access to partially dirty water<br>No access
	** **Presence of a clean water point?**	Yes, clean	Yes but partially dirty	Yes but dirty, or none

Category	Question	'Green' responses	'Amber' responses	'Red' responses
Working conditions	Number of hours working per day (on average)?	Up to 6 hours	More than 6, less than or equal to 9 hours	more than 9 hours
	Number of working days per week (on average)?	5 days or less	6 days	7 days
	At what age did the equid start to work?	Aged between 4 and 20 years old<br>greater than 5 years old (adult)	Between 1 and 3 years old<br>Older than 20 years	One year old or younger
	Does the equid get a rest break during the day?	yes, saddle/harness and load/vehicle removed	yes, saddle/harness and load/vehicle not removed	no rest
Housing conditions	Please indicate the housing regime of the equid	stabled equid with access to an exercise area<br>stabled equid with access to field<br>kept outside with access to shelter	indoor housing (stabled equid)	kept outside without access to shelter
	Is there a clean, dry lying area/comfortable resting area?	yes		No
	Is the environment clean?	yes		No
	Is the environment free from hazards?	yes		No

Table S2: Foal-specific questions and responses for the welfare categories of nutrition and behaviour. The questions indicated with asterisks represent the main question in the decision tree (*), and an alternative question (**) for conditions where it is not possible to answer the main question.

Category	Question	'Green' responses	'Amber' responses	'Red' responses
Nutrition	Body condition?	ideal	thin/moderate<br>fat	very thin/poor<br>very fat/obese
	Describe the foals access to milk	Free access to equid milk;	Free access to milk replacer (formulated for equids);	Limited access to equid milk; free access to any other non-equine specific milk
	How many hours per day does the foal spend with its mother?	Greater than 18 hours	12 to 18 hours	Less than 12 hours
	* Access to clean water during working period	free access to clean water	free access to partially dirty water<br>limited access to clean water	Free access to dirty water<br>limited access to partially dirty water<br>No access
	** Presence of a clean water point?	Yes, clean	Yes but partially dirty	Yes but dirty, or none
Behaviour	General attitude of the equid at a distance?	at ease - relaxed, calm and/or resting<br>alert and actively interested in surroundings		apathetic, depressed, withdrawn<br>agitated, aggressive, hyper-reactive/vigilant
	Please indicate signs of fear and distress present	no signs of fear and distress present		showing the whites of the eyes<br>unpredictable or sudden movements<br>sudden startle responses when standing quietly<br>aggressive behaviour<br>trembling or head shyness<br>completely withdrawn/shut down
	Is the foal showing signs of pain related behaviour?	no		yes
	* Owner's/user's/handler's interaction when holding the equid?	relaxed and confident	assertive / indifferent<br>cautious/fearful	aggressive
	** Presence of signs of harmful practices?	no		signs of hot brand, firing<br>signs of limb tethering or hobbling<br>signs of amputations or mutilations<br>signs of use of live serreta or similar

Table S3: Role of the equids assessed in each region and country. For working animals, the percentage of equids found in each place or type of work is provided. The percentage (Percent of equids) and number (n) of equids in each specified role per country, and in total, is provided

	**Countries**	**Equids role**	**Type of work (if applicable)**	**%**	**(n)**
**Asia**	China	Production animal	--	100	(61)
	India	Draft or pack animal	Brick kiln transport	85	(88)
			Construction site (pack)	15	(15)
	Nepal	Draft or pack animal	Brick kiln transport	95	(2434)
			Mountain transport (pack)	5	(123)
	Pakistan	Draft or pack animal	Brick kiln transport (pack)	24	(153)
			Coal mine transport (pack)	3	(20)
			Farm work	4	(23)
			Transport of goods to market (draft)	55	(347)
			Other work (pack or draft)	14	(92)
	Total	*Production animal*		*2*	*(61)*
		Draft or pack animal		*98*	*(3295)*
		Companion / sanctuary animal		*0*	*(0)*
**Europe**	Cyprus	Companion / sanctuary animal	--	100	(52)
	Greece	Draft or pack animal	Construction site (pack)	2	(5)
			Tourism (riding)	90	(18)
		Companion / sanctuary animal	--	8	(208)
	Ireland	Companion / sanctuary animal	--	100	(70)
	Italy	Production animal	--	81	(97)
		Companion / sanctuary animal	--	19	(23)
	Romania	Companion / sanctuary animal	--	94	(34)
		Draft or pack animal	Transport of goods to market	6	(2)
	Spain	Other work	Religious festival	100	(38)
	United Kingdom	Companion / sanctuary animal	--	100	(2658)
	Total	*Production animal*		*3*	*(97)*
		Draft or pack animal		*7*	*(215)*
		Companion / sanctuary animal		*89*	*(2855)*
	**Total**	**Production animal**		**2**	**(158)**
		**Draft or pack animal**		**53**	**(3510)**
		**Companion / sanctuary animal**		**44**	**(2855)**

Table S4: Percentage (%) and number (n) of each species of equids included in the WAG protocol assessment per country. Hybrids refers to hinnies and mules.

		Donkey		Hybrid		Horse	
	**Countries**	**%**	**(n)**	**%**	**(n)**	**%**	**(n)**
Asia	China	97	(59)	3	(2)	--	
	India	71	(73)	25	(26)	4	(4)
	Nepal	3	(83)	58	(1481)	39	(993)
	Pakistan	73	(466)	12	(75)	15	(94)
	Total	*20*	*(681)*	*47*	*(1584)*	*33*	*(1091)*
Europe	Cyprus	100	(52)	--		--	
	Greece	23	(53)	75	(173)	2	(5)
	Ireland	90	(63)	10	(7)	--	
	Italy	100	(120)	--		--	
	Romania	100	(36)	--		--	
	Spain	3	(1)	97	(37)	--	
	UK	91	(2427)	8	(203)		
	Total	*86*	*(2752)*	*13*	*(420)*	*< 1*	*(33)*
	**Total**	**53**	**(3433)**	**31**	**(2004)**	**17**	**(1124)**

Table S5: Percentage (%) and number (n) of each sex of equids included in the WAG protocol assessment per country. For a small number of animals, it was not possible to identify the sex for health and safety reasons.

		Female		Male		*Not assessed or not available*	
	**Countries**	**%**	**(n)**	**%**	**(n)**	*%*	*(n)*
Asia	China	80	(49)	20	(12)		--
	India	18	(19)	80	(82)	*2*	*(2)*
	Nepal	28	(722)	72	(1834)	*< 1*	*(1)*
	Pakistan	18	(113)	82	(522)		--
	Total	*27*	*(903)*	*73*	*(2450)*	*< 1*	*(3)*
**Europe**	Cyprus	31	(16)	69	(36)		--
	Greece	68	(156)	31	(71)	*2*	*(4)*
	Ireland	33	(23)	67	(47)		--
	Italy	73	(87)	24	(29)	*3*	*(4)*
	Romania	39	(14)	61	(22)		--
	Spain	37	(14)	63	(24)		--
	United Kingdom	33	(865)	67	(1792)	*< 1*	*(1)*
	Total	*37*	*(1175)*	*63*	*(2021)*	*< 1*	*(9)*
	Total	32	(2078)	68	(4471)	*< 1*	*(12)*
